# Transconjunctival Compression Sutures for Postoperative Hypotony After PRESERFLO MicroShunt Revision: A Case Report

**DOI:** 10.7759/cureus.102808

**Published:** 2026-02-02

**Authors:** Yudai Sato, Hidekazu Inami, Taiga Inooka, Ryo Tomita, Kenya Yuki

**Affiliations:** 1 Ophthalmology, Nagoya University Graduate School of Medicine, Nagoya, JPN

**Keywords:** bleb revision, exfoliation glaucoma, postoperative hypotony, preserflo microshunt, preserflo microshunt complications, transconjunctival compression sutures

## Abstract

This is a case in which postoperative hypotony following bleb revision after PRESERFLO^TM^ MicroShunt (MicroShunt) implantation in exfoliation glaucoma was successfully managed using transconjunctival compression sutures. An 82-year-old man with exfoliation glaucoma underwent MicroShunt implantation in the right eye for uncontrolled intraocular pressure (IOP). Due to bleb failure, open bleb revision with mitomycin C was subsequently performed. On postoperative day 14, severe hypotony with circumferential kissing choroidal detachment developed, and transconjunctival compression sutures were placed on the distal tube segment posterior to the MicroShunt fin to reduce aqueous outflow. Following the procedure, IOP gradually increased from 6 mmHg to 10 mmHg, accompanied by complete resolution of the choroidal detachment. At two months of follow-up, IOP remained stable without glaucoma medications. Transconjunctival compression suturing may represent a simple and minimally invasive treatment option for managing postoperative hypotony after MicroShunt implantation.

## Introduction

The PRESERFLO^TM^ MicroShunt (MicroShunt) is a minimally invasive surgical device designed to achieve long-term intraocular pressure (IOP) reduction in patients with glaucoma inadequately controlled by medical or laser therapy [1]. The device is made of biocompatible and inert poly(styrene-block-isobutylene-block-styrene), which minimizes tissue reaction and enables stable aqueous outflow. The MicroShunt is implanted using an ab externo approach, creating a controlled conduit that diverts aqueous humor from the anterior chamber to the subconjunctival space, resulting in the formation of a posterior, low-lying filtering bleb.

MicroShunt is designed to maintain a relatively constant aqueous outflow based on the Hagen-Poiseuille equation and is therefore considered less likely to cause hypotony-related complications [2]. A previous randomized controlled trial reported that hypotony, defined as an IOP < 6 mmHg, was significantly more frequent in the trabeculectomy group than in the MicroShunt group (51.1% vs. 30.9%; p < 0.001) [3]. However, hypotony still occurs at a certain frequency after MicroShunt surgery and even after bleb revision for MicroShunt [4].

The incidence of hypotony after open bleb revision performed for IOP elevation following MicroShunt implantation remains insufficiently reported. Strzalkowska et al. described the outcomes of open bleb revision with mitomycin C for bleb fibrosis after MicroShunt implantation in 27 eyes and reported no cases of postoperative hypotony [5]. In contrast, Theilig et al. reported postoperative choroidal detachment in three of 23 eyes following bleb revision after MicroShunt surgery [4]. These findings suggest that hypotony-related complications, including choroidal detachment, remain a potential risk even after bleb revision in eyes undergoing MicroShunt surgery.

Several treatment options have been reported for hypotony and choroidal detachment following MicroShunt implantation, including intraluminal stenting [6-9]. In contrast, for hypotony after trabeculectomy, additional approaches such as intracameral injection of balanced salt solution [10], viscoelastic agents [11], or 100% sulfur hexafluoride gas [12], and surgical drainage of suprachoroidal fluid [13] have been described. However, standardized management strategies for hypotony after MicroShunt implantation remain lacking.

Given that intraluminal stenting restores IOP by mechanically narrowing the MicroShunt lumen to reduce aqueous outflow without complete occlusion, we hypothesized that external compression of the device could achieve a similar effect by reducing luminal outflow. Based on this concept, we considered that compression with 8-0 Vicryl sutures could achieve a comparable degree of effective luminal narrowing. Because this approach aims to modulate, rather than eliminate, MicroShunt outflow and can be performed in a minimally invasive manner, we considered it an appropriate first-line surgical option before more invasive interventions. Herein, we report a case of exfoliation glaucoma that highlights the novelty and clinical relevance of transconjunctival compression sutures applied to the distal MicroShunt segment as a minimally invasive option for postoperative hypotony management.

## Case presentation

An 82-year-old man with exfoliation glaucoma underwent MicroShunt implantation for uncontrolled IOP in the right eye. His postoperative course was initially uneventful, and he was followed by a local ophthalmologist. He was later referred to our hospital because of elevated IOP in the right eye.

At presentation, the best-corrected visual acuity was 1.7 logMAR in the right eye and 0.1 logMAR in the left eye. IOP measured by Goldmann applanation tonometry was 26 mmHg in the right eye and 15 mmHg in the left eye. Bleb failure after MicroShunt implantation was diagnosed, and bleb revision surgery was performed.

The procedure was conducted in the operating room under local anesthesia. After ocular surface disinfection with povidone-iodine and sterile draping, a 6-0 silk traction suture was placed on the cornea to achieve downward gaze. Vertical and limbal conjunctival incisions were made at the site of the previous surgery, following the same approach as the initial procedure. The MicroShunt was identified, and the surrounding fibrotic tissue was carefully dissected using spring scissors. A sponge soaked in 0.04% mitomycin C was placed around the MicroShunt and within the subconjunctival space for five minutes, after which the area was thoroughly irrigated with 100 mL of balanced salt solution. The conjunctiva was closed with two interrupted 8-0 Vicryl sutures at the limbus, and the absence of wound leakage was confirmed. A subconjunctival injection of betamethasone sodium phosphate 2 mg (0.4%) was administered, and ofloxacin 0.3% ophthalmic ointment was applied at the end of the procedure. Postoperatively, topical levofloxacin 1.5% eye drops and betamethasone sodium phosphate 0.1% eye drops were prescribed four times daily.

On postoperative day 1, the IOP was 5 mmHg, the anterior chamber depth was deep, and no obvious choroidal detachment was observed on fundus examination. A low-lying filtering bleb was noted.

**Figure 1:**
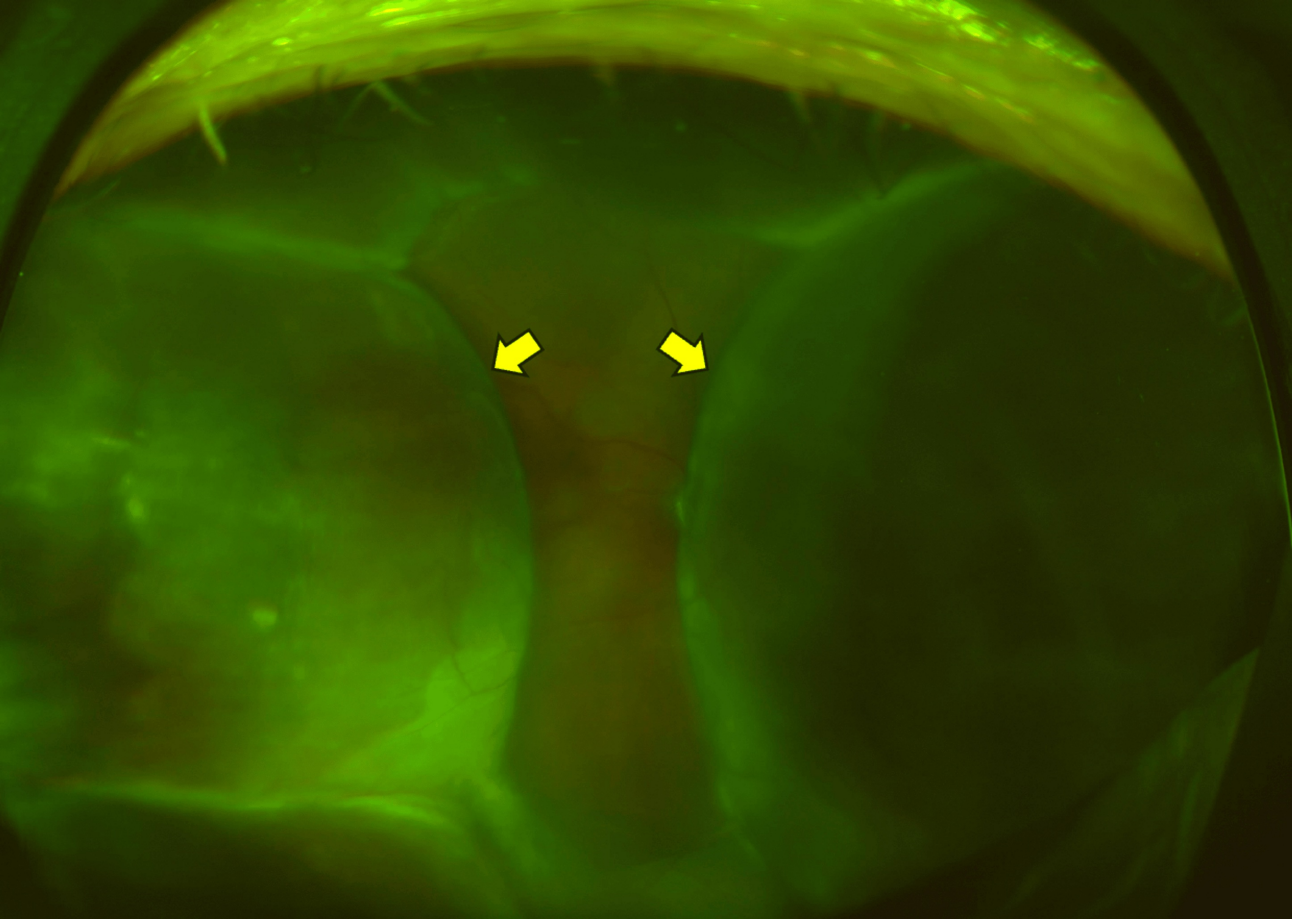
Fundus photograph taken two days after bleb revision surgery This fundus photograph, taken two days after the bleb revision surgery, shows a 360-degree choroidal detachment. The yellow arrows indicate choroidal detachment

On postoperative day 2, the IOP further decreased to 3 mmHg, and a mildly shallow anterior chamber was observed. The height of the filtering bleb remained largely unchanged. Fundus examination revealed circumferential choroidal detachment sparing the posterior pole (Figure 1). We added topical 1% atropine sulfate hydrate eye drop once daily. The topical steroid regimen was maintained with 0.4% betamethasone eye drops four times daily, and no subconjunctival steroid injections or systemic prednisolone were administered.

On postoperative day 7, there were no significant changes in the clinical findings; the IOP was 3 mmHg, with a mildly shallow anterior chamber and choroidal detachment of similar extent.

**Figure 2:**
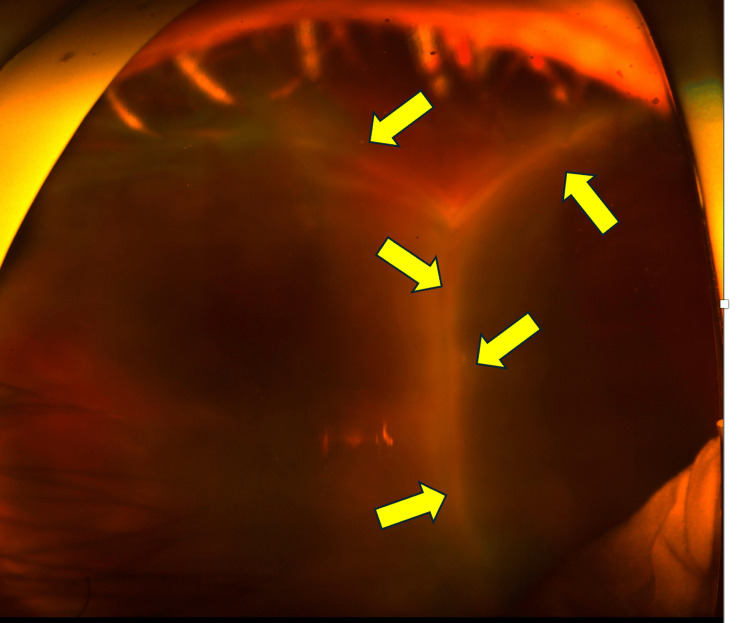
Fundus photograph taken 14 days after bleb revision surgery This fundus photograph, taken 14 days after the bleb revision surgery, shows a 360-degree kissing choroidal detachment. The yellow arrows indicate kissing choroidal detachment

On postoperative day 14, the IOP was 6 mmHg, and although the anterior chamber depth showed no significant change, circumferential kissing choroidal detachment was observed (Figure 2). A low-lying, flat filtering bleb was observed. In this case, the treating surgeon is a glaucoma specialist and does not perform vitrectomy. However, we are familiar with and capable of managing kissing choroidal detachment, including choroidal drainage when indicated. Therefore, a vitreoretinal surgical consultation was not obtained. In this case, choroidal drainage was not performed because aqueous overfiltration was considered the primary pathogenic mechanism, and there were no clinical signs of suprachoroidal hemorrhage. Accordingly, management was directed toward reducing aqueous outflow rather than proceeding with surgical drainage.

**Figure 3:**
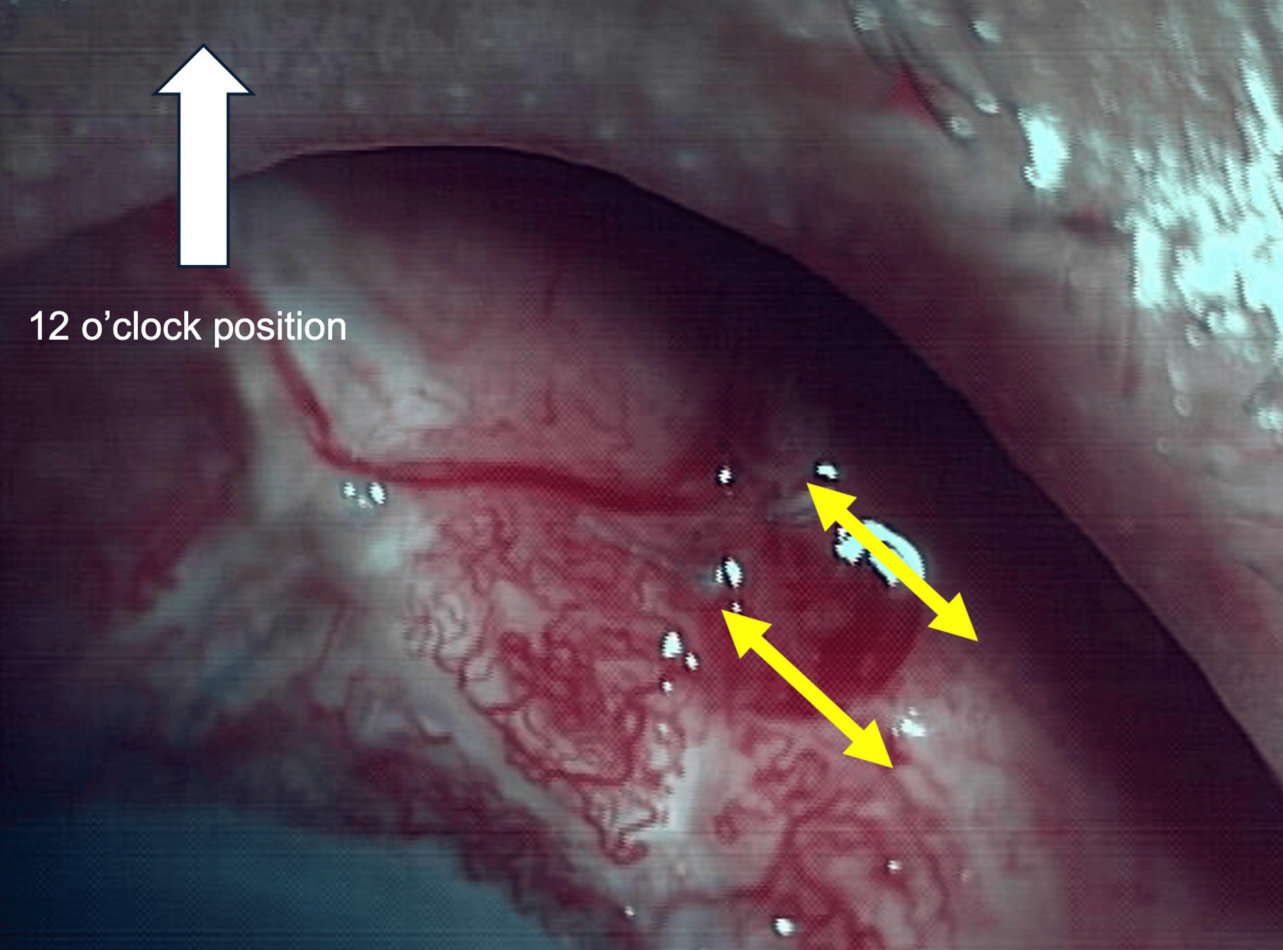
Two transconjunctival scleral compression sutures were placed with 8-0 Vicryl Two transconjunctival scleral compression sutures were placed with 8-0 Vicryl to compress the posterior portion of the MicroShunt. The arrows indicate the 8-0 Vicryl sutures. This image was captured immediately after the compression suture was placed

On the same day, transconjunctival compression suturing of the tube posterior to the fin of the MicroShunt was performed (Figure 3). Intervention was undertaken on postoperative day 14 because circumferential kissing choroidal detachment was observed at that time. Kissing choroidal detachment is generally considered an indication for urgent surgical intervention [13]. 

**Figure 4:**
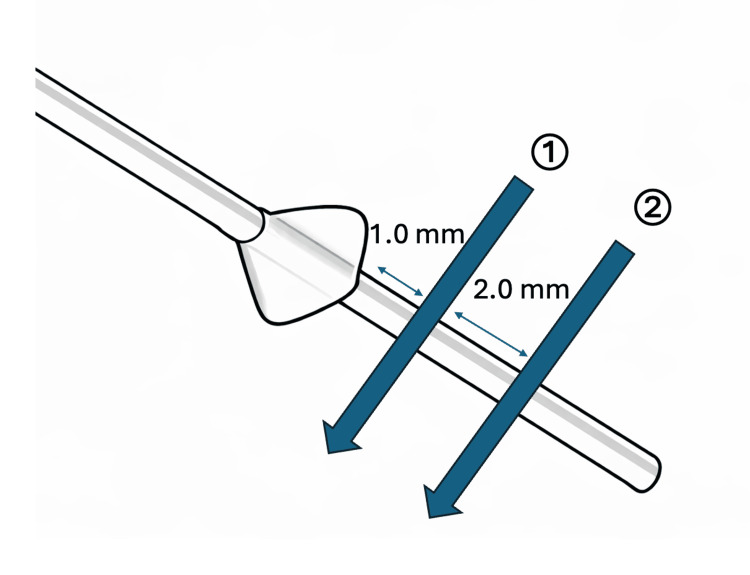
Schematic illustration of the detailed suture placement The first compression suture was placed approximately 1.0 mm posterior to the estimated fin edge, with the needle passed relatively deep to ensure secure encirclement of the MicroShunt. The second suture was placed in a similar manner approximately 3.0 mm posterior to the fin edge and tied with firm tension

In the outpatient procedure room, after disinfection with povidone-iodine and with the patient instructed to gaze downward, two tight transconjunctival scleral sutures using 8-0 Vicryl were placed from the fin to the posterior end of the MicroShunt to achieve mechanical compression of the device (Figure 4).

The filtering bleb was relatively flat, and the sutures were placed without intentional bleb compression. Because thick Tenon’s tissue overlying the MicroShunt made direct visualization difficult, the posterior edge of the fin was estimated using anatomical landmarks, defined as approximately 4 mm posterior to the limbus. Allowing for a safety margin, two compression sutures were placed posterior to the estimated fin edge with sufficient depth to encircle the MicroShunt (Figure 4). 

Follow-up earlier than one week after the procedure was not feasible due to a lack of family support; therefore, the postoperative assessment was scheduled at one week. Changes in choroidal detachment were evaluated by acquiring ultra-widefield fundus images using Optos imaging at each follow-up visit, which allowed consistent and objective assessment of the extent of choroidal detachment and its partial resolution.

**Figure 5:**
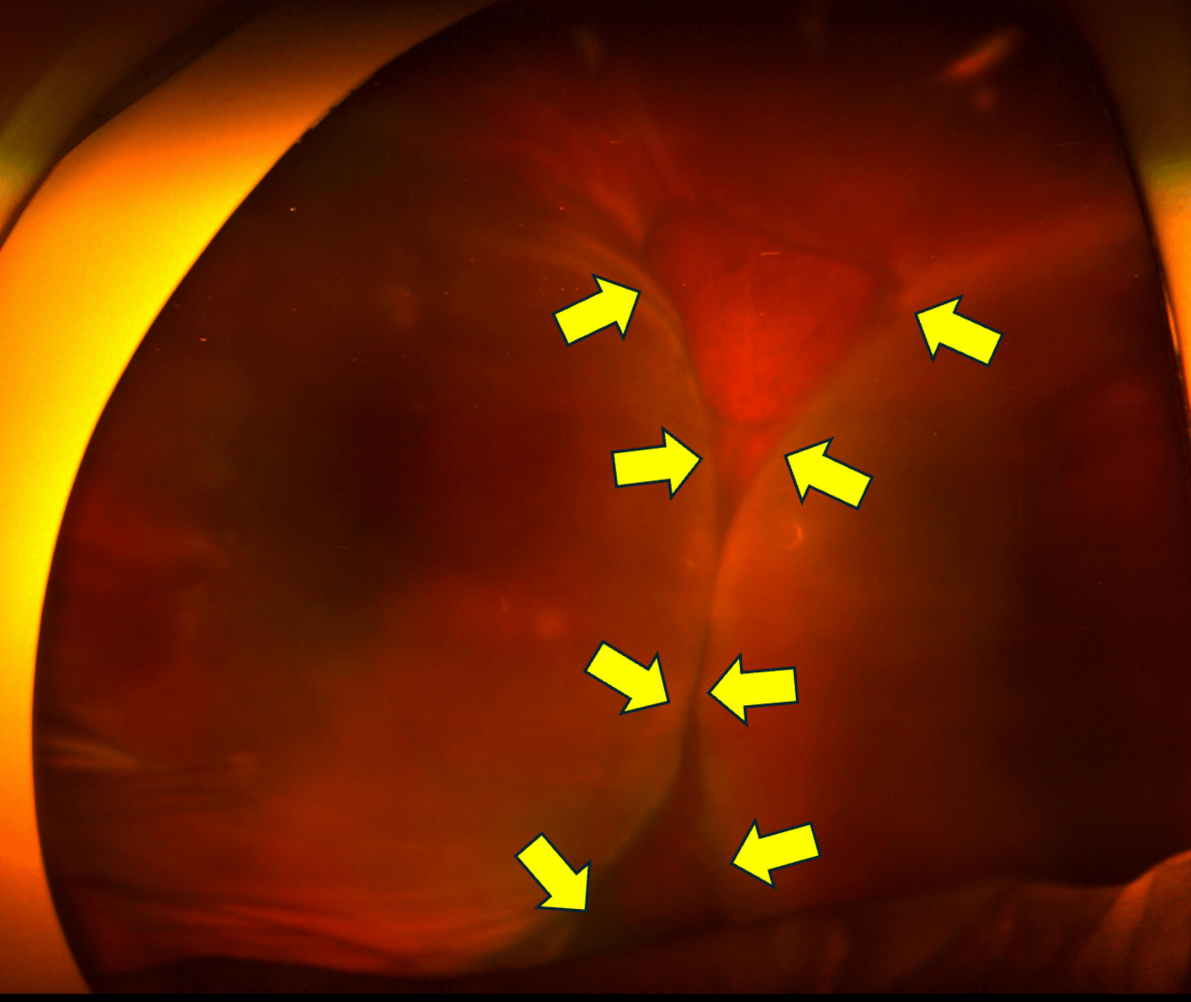
This fundus photograph, taken one week after the compression sutures This fundus photograph, taken one week after the compression sutures, shows improvement of the choroidal detachment. A reduction in kissing choroidal detachment was observed; the areas of choroidal detachment are indicated by yellow arrows

On postoperative day 7 after compression suturing (postoperative day 21 after bleb revision), IOP increased to 8 mmHg. The choroidal detachment had decreased superiorly and inferiorly. Although the nasal and temporal choroidal detachments were kissing at their highest points, the overall extent had diminished (Figure 5). The anterior chamber depth had returned to normal. 

**Figure 6:**
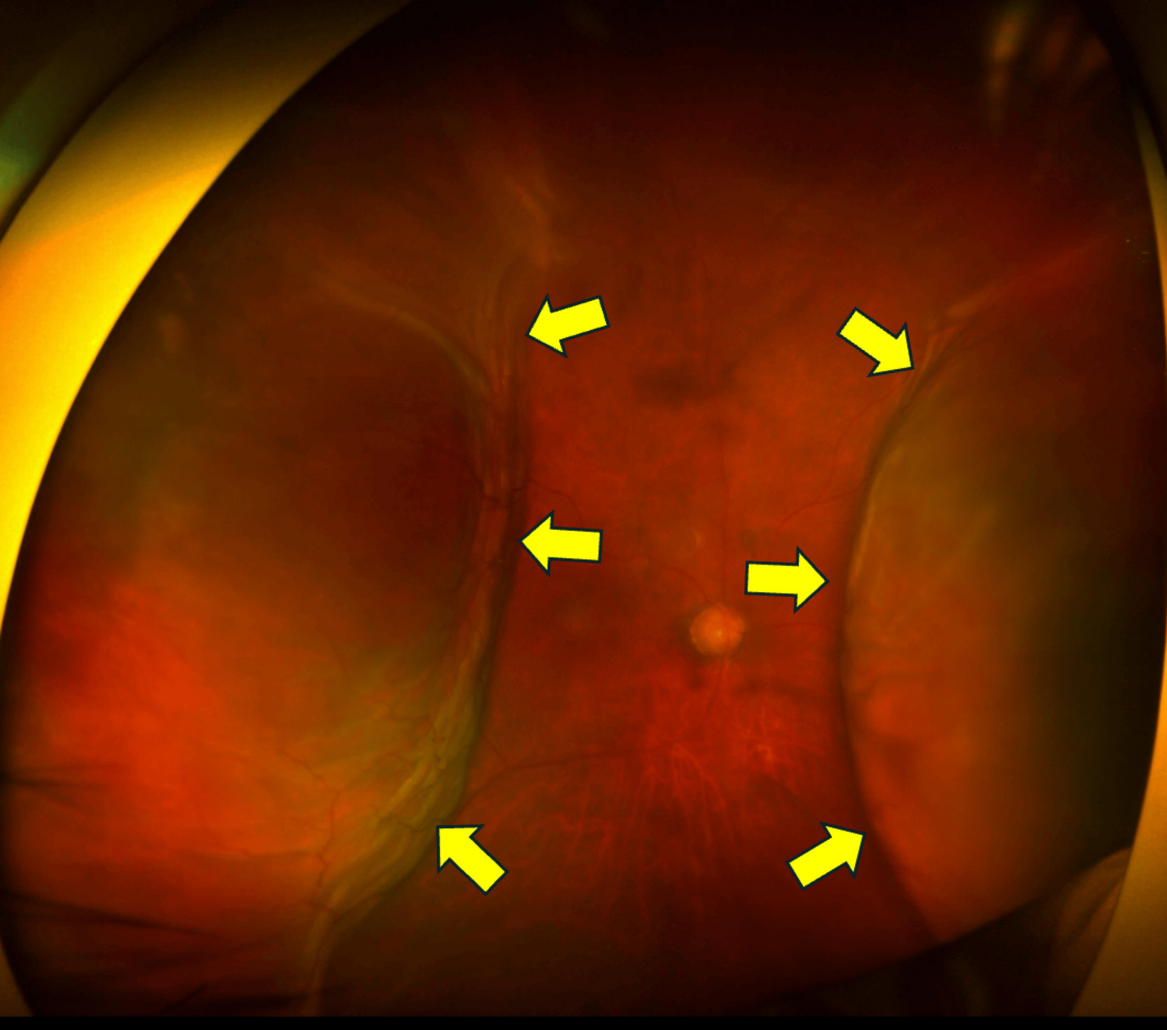
Fundus photograph, taken two weeks after the compression sutures This fundus photograph, taken two weeks after the compression sutures, demonstrates partial resolution of the choroidal detachment. The yellow arrows indicate the margin of the choroidal detachment

On postoperative day 14 after compression suturing (postoperative day 28 after bleb revision), the IOP increased to 10 mmHg, and the superior and inferior choroidal detachments had resolved. Although nasal and temporal choroidal detachments persisted, contact between the detachments had resolved, allowing visualization of the posterior pole (Figure 6). The filtering bleb had become flat.

**Figure 7:**
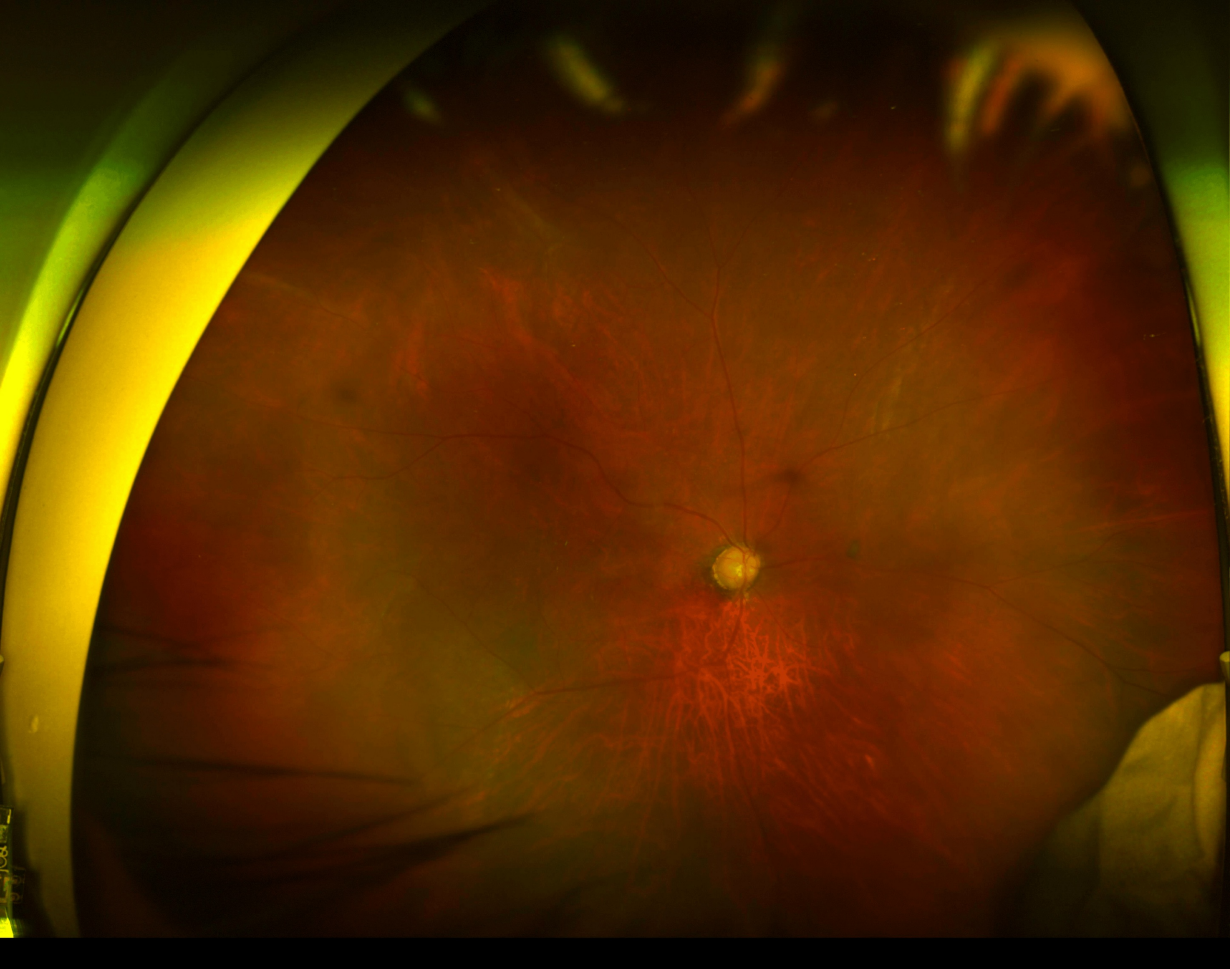
Fundus photograph, taken three weeks after the compression sutures This fundus photograph, taken three weeks after the compression sutures, demonstrates complete resolution of the choroidal detachment

On postoperative day 21 after compression suturing (postoperative day 35 after bleb revision), the nasal and temporal choroidal detachments had also resolved (Figure 7). The anterior chamber depth was normal, the filtering bleb remained flat, and the IOP was 10 mmHg without the use of anti-glaucoma medications. 

On postoperative day 35 after compression suturing (postoperative day 49 after bleb revision), the IOP was 10 mmHg without the use of antiglaucoma medications. The choroidal detachment had completely resolved.

On postoperative day 49 after compression suturing (postoperative day 63 after bleb revision), the IOP was maintained at 10 mmHg without the use of antiglaucoma medications; however, the filtering bleb was not clearly identifiable. The patient was then referred back to his primary ophthalmologist. At the final follow-up visit, the best-corrected visual acuity was 2.3 logMAR. The clinical course of this case is summarized in Table 1.

**Table 1:** Postoperative clinical course and outcomes of this patient POD: postoperative day after bleb revision; IOP: intraocular pressure; BCVA: best-corrected visual acuity; Log MAR: logarithm of the minimum angle of resolution; levofloxacin: levofloxacin 1.5% eye drops; betamethasone: betamethasone sodium phosphate 0.1% eye drops; atropine: atropine sulfate hydrate 1% eye drops

POD	Bleb revision	1	2	14	Compression suture	21	28	35	49
IOP (mmHg)	26	5	3	6		8	10	10	10
BCVA (Log MAR)	1.7								2.3
Levofloxacin		4	4	4		4	4		
Betamethasone		4	4	4		4	4	4	4
Atropine			1	1		1	1	1	
Fundus image			Figure 1	Figure 2	Figures 3-4	Figure 5	Figure 6	Figure 7	

## Discussion

In this report, we describe a case in which postoperative hypotony with choroidal detachment following bleb revision after MicroShunt implantation was successfully managed using transconjunctival compression sutures on the tube posterior to the fin of the MicroShunt. Although the MicroShunt procedure is generally associated with a lower risk of hypotony than trabeculectomy, our findings demonstrate that hypotony-related complications can still occur, even after bleb revision following MicroShunt implantation.

The present patient was an 82-year-old man with exfoliation glaucoma, a population known to be at higher risk for postoperative hypotony. Nasyrov et al. evaluated the incidence, outcomes, and risk factors for hypotony and choroidal detachment following standalone PRESERFLO MicroShunt implantation and found significantly higher incidences of hypotony (83.7% vs. 69.4%, p < 0.05) and choroidal detachment (34.6% vs. 20.6%, p < 0.05) in the eyes with exfoliation glaucoma [14]. Identified risk factors included exfoliation glaucoma, hyperopia, lower preoperative IOP, greater postoperative IOP reduction, male sex, older age, and a higher preoperative medication burden. The present case involved an elderly male patient with exfoliation glaucoma, placing him at a particularly high risk for hypotony-related complications even after bleb revision of the MicroShunt.

Management of choroidal effusions typically begins with medical therapy but may require procedural or surgical intervention. Because hypotony, particularly after glaucoma surgery, is a key contributor to effusion development, interventions aimed at increasing IOP should be considered. These include anterior chamber injection of balanced salt solution [10], a cohesive viscoelastic agent [11], or 100% sulfur hexafluoride gas [12] and surgical drainage of suprachoroidal fluid [13]. However, as with any surgical procedure, it carries a greater risk than nonsurgical management, prompting ongoing efforts to reduce morbidity through the development of less invasive techniques.

Intracameral injection of balanced salt solution or a viscoelastic agent is a potential option for increasing IOP. However, at our institution, any intraocular intervention involving anterior chamber manipulation must be performed under hospitalization. In addition, the use of balanced salt solution or viscoelastic agents for this indication is not covered by insurance and entails substantial additional cost. Furthermore, intracameral injection of a viscoelastic agent for the treatment of hypotony represents an off-label use in Japan, limiting the practicality of this approach in our clinical setting. For these reasons, compression suturing was selected as the initial intervention.

At our institution, choroidal drainage requires inpatient admission, and in the present case, the patient would have needed to wait approximately one week before hospitalization. Moreover, choroidal drainage is an invasive procedure. A prospective randomized study evaluating management strategies for flat anterior chamber with choroidal detachment after trabeculectomy reported that anterior chamber reformation combined with choroidal drainage was associated with a significantly higher risk of postoperative visual acuity decline compared with medical therapy alone [15].

Intraluminal stenting has been reported as an effective treatment option for hypotony following MicroShunt implantation [6-9]. Kitamura et al. reported a case of severe hypotony with choroidal detachment after bilateral PRESERFLO MicroShunt implantation, in which ab externo intraluminal insertion of a 9-0 nylon suture successfully increased IOP and resolved the detachment [6]. Miura et al. described successful ab interno intraluminal stenting using a 10-0 nylon suture for prolonged hypotony following MicroShunt implantation, leading to rapid normalization of IOP and resolution of hypotony-associated complications [7]. Aguilar-Muñoa et al. also reported successful treatment of hypotony in six of seven cases using ab externo intraluminal insertion of a 9-0 polypropylene suture [9].

Despite its effectiveness, intraluminal stenting has several limitations. The ab externo approach requires conjunctival opening, which may promote scarring of the filtering bleb, whereas the ab interno approach avoids conjunctival dissection but requires corneal incisions, is technically more demanding, and carries a small but potential risk of endophthalmitis. In contrast, transconjunctival compression suturing is a simple, minimally invasive, and easily performed technique that does not require conjunctival opening or intraocular manipulation. Therefore, this approach may represent a reasonable first-line option before attempting more invasive interventions.

We selected 8-0 Vicryl as an absorbable suture with the expectation that, after resolution of the choroidal detachment, gradual suture absorption would allow spontaneous reformation of the filtering bleb without the need for additional intervention. However, we acknowledge that absorbable polyglactin sutures can induce conjunctival inflammation or granulomatous reactions, which could adversely affect bleb function. In future similar cases, the use of a nonabsorbable suture such as nylon should be considered to minimize inflammation-related risks.

The image showing marked conjunctival hyperemia was obtained immediately after placement of the compression sutures and therefore is unlikely to represent an inflammatory response to the 8-0 Vicryl suture material itself. However, we acknowledge that, over the longer term, inflammation associated with absorbable sutures such as 8-0 Vicryl could contribute to subconjunctival fibrosis and scarring, potentially increasing the risk of late bleb dysfunction. This consideration reflects a potential trade-off between short-term resolution of hypotony and long-term bleb survival. Accordingly, when using Vicryl sutures for this technique, the possibility of compromised long-term bleb function should be carefully considered.

In the present case, marked conjunctival hyperemia was observed, suggesting a potential risk of future bleb dysfunction. In such situations, enhanced anti-inflammatory management, such as subconjunctival injections of 5-fluorouracil or antivascular endothelial growth factor agents, as well as sub-Tenon’s injection of triamcinolone in combination with compression sutures, may be beneficial for maintaining bleb function. In addition, the use of a nonabsorbable suture material, such as nylon, should be considered in future cases to reduce inflammation-related risks.

Because compression sutures are placed transconjunctivally in close proximity to an active aqueous outflow pathway, the risk of infection cannot be completely excluded. Suturing directly over the MicroShunt tube may theoretically pose a higher risk of blebitis or endophthalmitis than ab externo or ab interno intraluminal stenting, as the latter does not involve transconjunctival suture tracts adjacent to functioning aqueous flow. Therefore, meticulous antiseptic preparation before suturing, postoperative prophylactic topical antibiotics, and careful patient education regarding early signs of infection, such as ocular discharge, pain, or increasing conjunctival hyperemia, are essential when employing this technique.

This report has several limitations. First, it describes a single case, which limits the generalizability of the findings. Second, because the technique is performed in a blind manner, it remains uncertain whether the MicroShunt lumen is consistently narrowed as intended. Third, there is a potential risk of leakage from the suture entry site. Fourth, in the present case, marked conjunctival hyperemia and ciliary injection were observed after bleb revision, raising the possibility that local conjunctival inflammation induced by the absorbable Vicryl sutures contributed to a transient reduction in aqueous outflow through inflammation-related wound healing. Accordingly, the relative contribution of mechanical compression versus inflammation-related flow reduction cannot be definitively determined. This limitation should be considered when interpreting the results and may have implications for long-term bleb survival. Fifth, because long-term follow-up data after complete absorption of the Vicryl sutures were not available, the long-term stability of IOP following transconjunctival compression suturing could not be fully evaluated. In addition, spontaneous resolution cannot be completely excluded, as the choroidal detachment resolved approximately five weeks after bleb revision in this case. Further accumulation of cases and systematic evaluation are warranted to better define the safety, efficacy, and optimal indications of this technique.

## Conclusions

Postoperative hypotony with choroidal detachment can occur even after bleb revision following PRESERFLO™ MicroShunt implantation, particularly in high-risk patients such as elderly individuals with exfoliation glaucoma. Transconjunctival compression suturing of the MicroShunt represents a simple, minimally invasive, and effective treatment option for managing hypotony-related complications. This technique may be considered as a first-line intervention before more invasive procedures, including intraluminal stenting or choroidal drainage, are undertaken.
